# A pilot study of a joint outdoor exercise program for dog owners and dogs

**DOI:** 10.1038/s41598-024-65033-0

**Published:** 2024-06-21

**Authors:** K. Smedberg, E. Lundbeck, E. Roman, J. W. Eriksson, S. Spörndly-Nees, L. V. Kallings, A. Bergh, J. Söder

**Affiliations:** 1 Department of Animal Biosciences, Swedish University of Agricultural Sciences, Uppsala, Sweden; 2 Department of Pharmaceutical Biosciences, Uppsala University, Uppsala, Sweden; 3 Department of Medical Sciences, Uppsala University, Uppsala, Sweden; 4 Department of Women’s and Children’s Health, Physiotherapy and Behavioural Medicine, Uppsala University, Uppsala, Sweden; 5 Primary Care and Health, Region Uppsala, Uppsala, Sweden; 6 Department of Physical Activity and Health, The Swedish School of Sport and Health Sciences, Stockholm, Sweden; 7 Family Medicine and Preventive Medicine, Department of Public Health and Caring Sciences, Uppsala University, Uppsala, Sweden; 8 Department of Clinical Sciences, Swedish University of Agricultural Sciences, Uppsala, Sweden

**Keywords:** Medical research, Quality of life, Health policy, Lifestyle modification

## Abstract

Increasing levels of physical inactivity is a global burden for mankind and is also an emerging problem in companion dogs. In both humans and dogs, insufficient physical activity is associated with increased risk for noncommunicable diseases and impaired quality of life (QoL). The aim of the current pilot study was to evaluate effects of a joint outdoor exercise program for dog owners (n = 22) and dogs (n = 22) with focus on QoL and body measurements. Results indicate that an eight-week exercise intervention alone, with a target distance of at least 2 km twice a week, may be sufficient to significantly increase self-reported QoL and acceptance of bodily appearance in dog owners despite no reductions in body measurements. In dogs, a significantly reduced body condition score (BCS) was registered, despite no considerable changes in feeding. The increased owner motivation for continued joint exercise suggests potential for lifestyle changes, which could be investigated in future studies including control groups and long-term follow-ups. The importance of the human-animal bond as a success factor for increased mutual physical activity and health benefits in both dog owners and dogs is recommended to be studied in a more in-depth manner.

## Introduction

As the world is recovering from the COVID-19 pandemic, the epidemic of physical inactivity remains a global threat. Insufficient physical activity is classified as the fourth leading risk factor for mortality and constitutes a well-known risk factor for major noncommunicable diseases such as ischemic heart disease, type 2 diabetes and colon and breast cancer^1–3^. Even though evidence for the benefits of physical activity has been available for decades, 1.4 billion adults (27.5% of the adult population) do not meet the recommendations for physical activity according to the latest global estimates from 2016^4^. Insufficient physical activity is also an emerging problem for our companion dogs^5,6^. Even though several studies have shown that dog ownership is associated with higher levels of physical activity^6–10^, up to 40% of Swedish dogs are overweight and similar numbers have been reported from other western countries^11–13^. As in humans, overweight in dogs is mostly caused by positive energy balance due to a lack of physical activity or excessive energy intake^6^. The consequences of overweight in dogs mimic the ones in humans, with increased risk for metabolic disturbances and chronic diseases^14–16^.

Apart from the increased risk for noncommunicable diseases, physical inactivity also has negative effects on mental health and quality of life (QoL)^4^. Consequently, increased physical activity has been shown to increase mental wellbeing and QoL in human^17,18^. In this respect, it has been argued that physical activity undertaken outdoors is preferable, since it is associated with greater reductions of stress and depressive symptoms and increased feelings of revitalization and positive engagement compared to indoor physical activity^19–21^. There is a lack of studies on how physical inactivity affects mental wellbeing in dogs, but it has been shown that obesity in dogs is associated with a reduction of QoL, which is improved after successful weight loss^22^.

In 2018, the World Health Organization (WHO) adopted the Global Action Plan on Physical Activity 2018–2030 (the GAPPA recommendations) to provide countries with a framework of effective and feasible policy actions to increase physical activity at all levels, stressing a strategic combination of “upstream” policy actions and “downstream” individually focused educational and informational approaches^23^. In their first Global status report on progress of the implementation of GAPPA recommendations, the WHO concluded that implementation so far has been slow and uneven^24^. One recommendation for the way forward that the report offers is to effectively engage nongovernmental actors and the community, and to build capacity in people. All countries are recommended to “test innovative solutions across diverse contexts”^24^.

The objective of the current study was to evaluate one such innovative solution; a joint outdoor exercise program for dog owners and dogs, originally designed by the Swedish Working Dog Association and funded by the Swedish Outdoor Association^25^, with the aim of increasing outdoor physical activity and improving health and wellbeing in both parties. One Health approaches to increase physical activity in both dog owners and dogs have been advocated in previous studies, as dog owners and dogs often have a shared lifestyle and mutual habits of physical activity^12,26,27^. As Sweden has approximately 800,000 registered dog owners and 1,100,000 dogs^28^, dog owners constitute a considerable target group for strategies to increase physical activity. In the current study, effects of the joint outdoor exercise program were evaluated on self-assessed physical activity in dog owners, owner-assessed QoL in dog owners and dogs and on different body measurements in both parties.

## Methods

### Ethical statement

The study was approved by the Ethics Committee for Animal Experiments, Uppsala, Sweden (Dnr 5.8.18-15533/2018) and by the Swedish Ethical Review Authority, Stockholm, Sweden (Dnr 2021-01014). It was conducted in accordance with the Declaration of Helsinki, the guidelines of the Swedish Legislation on Animal Experimentation (Animal Welfare Act SFS 2018:1192) and the European Union Directive on the Protection of Animals Used for Scientific Purposes (Directive 2010/63/EU). Informed written consent was obtained from all dog owners before entering the study.

### Study population

#### Dog owners

Dog owners were invited to participate in an intervention pilot study with a joint outdoor exercise program with their dogs, and voluntarily signed up for the study through an internet-based application form distributed through social media and on the websites of the Swedish Working Dog Association and the Swedish University of Agricultural Sciences (SLU). Participants were included on a non-randomized basis based on inclusion and exclusion criteria. The inclusion criteria were age ≥ 18 years and in physical and mental condition allowing participation at the minimum level in the exercise program. The exclusion criteria included diseases that could entail a risk when participating in the study, i.e. known ischemic heart disease or heart failure, active malignancy, type 1 diabetes, chronic pulmonary diseases or severe psychiatric disorders, including alcohol and substance use disorders.

#### Dogs

The inclusion criteria for participating dogs were age ≥ 1 year and in physical condition allowing participation at the minimum level in the exercise program. The exclusion criteria included known systemic or orthopedic disease that could entail a risk when participating in the study, or known aggressiveness or timidity that could affect the ability to be handled by researchers. All dogs participated in the study with their owner or handler.

### Study protocol

**Figure 1:**
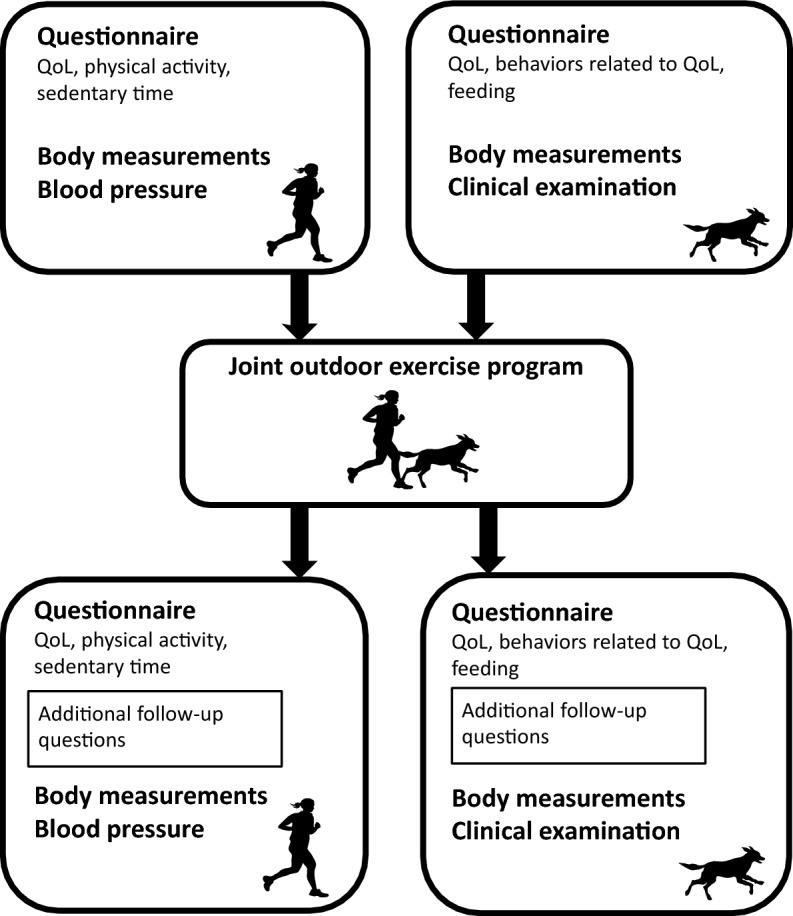
Experimental outline of the joint outdoor exercise program for dog owners and dogs. Questionnaires, body measurements and blood pressure were used as outcome measures before and after the intervention. Additional questions on perceived overall changes were included in the follow-up questionnaires. *QoL* quality of life.

The overall experimental outline is shown in Fig. 1. Dog owners and dogs participated in an eight-week joint outdoor exercise program designed by the Swedish Working Dog Association, with the aim of increasing physical activity in dog owners and dogs and thereby promoting health and wellbeing in both parties^25^. Before the start of the intervention, dog owners answered a questionnaire regarding their own self-assessed QoL, and another questionnaire regarding QoL and feeding routines/feeding-related behavior in their dogs. Dog owners were also asked to assess their own weekly physical activity and daily sedentary time. The same questionnaires were answered in the end of the last week of the exercise program (henceforth referred to as “after the intervention”), with additional questions on perceived overall changes in the assessed perceptions, behaviors or routines in dog owners and dogs. Both dog owners and dogs underwent body measurements before and after the intervention, and dogs underwent clinical examinations. For dog owners, blood pressure was measured before and after the intervention. All clinical evaluations were conducted in Uppsala, Sweden during August to October 2021.

#### Joint outdoor exercise program

The eight-week outdoor exercise program included joint jogging and circuit training sessions for dog owners and dogs. In order to be suitable for most dog owners and dogs, irrespective of previous exercise experience or physical capacity, four different target jogging levels were available; 2, 5, 7.5 or 10 km. Dog owners individually selected a suitable target jogging distance for themselves as well as for their dogs, with the goal of reaching the selected distance at the end of the eight week. Jogging sessions were performed twice a week, except for participants in the 10 km group, who performed three jogging sessions per week. Both distance and intensity were gradually increased throughout the eight weeks. Participants aiming for distances between 2 and 7.5 km were instructed to alternate between jogging and walking until the very last week of the exercise program. For participants aiming for 10 km, intensity was slowly increased throughout the program through interval sessions with gradually decreasing resting periods. Circuit training exercises designed for joint performance by dog owners and dogs were conducted once a week for all participants. Six different exercises were performed at each session; four strength exercises focusing on leg/hindleg, arms/foreleg, core and neck muscles and two exercises focusing on agility and explosive speed^29^. Time per exercise was gradually increased from 30 to 60 s during the program. Throughout the exercise program, it was optional to have the dog on or off leash.

### Outcome measures

#### Questionnaires

All questionnaires were designed in the digital software Netigate (Netigate AB, Stockholm, Sweden). A Likert scale of 1–5 was used in all questionnaires, except for time assessments of physical activity and sedentary behavior in owners and questions on feeding routines in dogs. The questionnaires were answered before the start of the joint outdoor exercise program and, as follow-up, in the last week of the program. The follow-up questionnaires contained additional questions on perceived overall changes in assessed perceptions, behaviors or routines in dog owners and dogs. The additional questions were constructed as statements in which dog owners were asked to evaluate a subjectively perceived change on a Likert scale—for example “I assess my QoL to be better now compared to before the joint exercise program”, “I assess my dog as more playful now compared to before the joint exercise program”. All questionnaires regarding dog owners and dogs are included in Supplementary Materials 1 (in Swedish) and in Supplementary Materials 2 (translated into English).

#### Dog owner questionnaires

The dog owner questionnaires consisted of questions on QoL, physical activity and sedentary time. For QoL assessments, eleven questions from the questionnaire the World Health Organization Quality of Life Brief Version (WHOQOL-BREF) were used^30^. These specific questions were selected since it was hypothesized that their outcome could be affected by physical activity, and because their Likert scale had a similar arrangement as the one used in the dog questionnaire (that is, the highest numbers represented “Very good” or “Completely” etc.). The overall focus of the selected questions was on QoL, enjoyment and energy for everyday life, satisfaction with one’s health, acceptance for bodily appearance, safety in daily life, opportunities for leisure activities and abilities to concentrate and get around physically. For assessment of time spent on physical activity per week, two questions designed by the Swedish National Board of Health and Welfare^31^ were used. These questions are used in the Swedish health care system to identify people that are insufficiently physically active, and for monitoring the level of physical activity post intervention. The questions distinguish between time spent on everyday physical activity, such as walking or gardening, and time spent exercising. The latter is defined as physical activities resulting in shortness of breath, such as jogging and other sport activities. For everyday physical activity, seven categories were presented; “0 min”, “ < 30 min”, “30–59 min”, “60–89 min”, “90–149 min”, “150–300 min” and “ > 300 min”. For exercise, six categories were presented; “0 min”, “ < 30 min”, “30–59 min”, “60–89 min”, “90–120 min”, and “ > 120 min”. For assessment of daily sedentary time (sleep excluded), the stationary single-item question developed by Kallings et al.^32^ was used. Seven categories were presented, ranging from “Virtually all day”, “13–15 h”, “10–12 h”, “7–9 h”, “4–6 h”, “1–3 h” to “Never”. The follow-up questionnaire contained two additional questions; one on perceived overall changes of QoL and one on motivation to exercise together with the dog. The follow-up questions were created by the authors of this study.

#### Dog questionnaires

The dog questionnaires consisted of three sections, which in all contained eleven question blocks. The first section consisted of one question block with three questions on overall QoL in dogs, created by the authors of this study with inspiration from questionnaires developed by WHO^30^ and Lavan^33^. In this section, dog owners were asked to evaluate three statements: “My dog has more good days than bad days”, “My dog likes its life” and “My dog has a good QoL” on a Likert scale from 1 (“totally disagree”) to 5 (“totally agree”). At follow-up, this section had one additional question on perceived overall changes in QoL, created by the authors of this study. The second section consisted of nine questions block with in total 76 questions assessing different canine behaviors related to QoL. The included blocks were "Exercise and obedience", "Fear and anxiety", "Curiosity", "Tendency to get excited or agitated", "Affection and contact-seeking", "Playfulness", "Social contact", "Passivity, rest and sleep" and "Vitality, activity and movement". The vast majority of the questions in this section was based on the Canine Behavioral Assessment and Research Questionnaire (C-BARQ) developed by Hsu and Serpell^34^, with slight modifications. Since known aggressiveness was an exclusion criterion for dogs participating in the study, C-BARQ-questions regarding aggression were omitted. In order to get a more comprehensive coverage of behaviors related to rest and activity, 16 questions in the blocks "Passivity, rest and sleep" and "Vitality, activity and movement" were designed by the authors. Inspiration was taken from questionnaires developed by Lavan (2013) regarding happiness and physical functioning^33^ and by German et al. (2012) regarding vitality^22^. At follow-up, all these question blocks had one additional question each on perceived overall changes within each behavior block, except for the block “Vitality, activity and movement” which had two additional questions; one on perceived subjective changes in vitality and one on perceived subjective changes in cardio fitness in the dog. The follow-up questions were created by the authors of this study, and consisted of statements that dog owners were asked to evaluate, such as “I assess my dog’s cardio fitness to be better now compared to before the joint exercise program”. The third section consisted of one question block with seven questions on feeding habits and feeding-related behaviors, such as tendency to beg for food. These questions were created by the authors with inspiration from questionnaires developed by Lavan (2013) regarding appetite^33^. At follow-up, this section had one additional question on perceived subjective overall changes in the dog’s appetite. This question was created by the authors of this study.

### Body measurements and blood pressure

#### Dog owners

Height was measured to the nearest mm with a seca 123 stadiometer (seca GmbH & Co., Hamburg, Germany), with the head positioned in a Frankfort plane with the lower eye socket in horizontal level with the upper ear canal^35^. Bodyweight was measured to the nearest hg with a Kruuse Scale 250 digital veterinary scale (Jørgen Kruuse A/S Langeskov, Denmark). Body mass index (BMI) was calculated as described by WHO^36^ by dividing body weight (kg) by height (m) squared. A BMI range of 18.5 to < 25 kg/m^2^ was considered normal, ≥ 25 to < 30 as overweight and ≥ 30 as obesity^36^. Waist-hip ratio (WHR) was calculated as described by WHO^37^ by dividing mean waist circumference (cm) by mean hip circumference (cm). Waist and hip circumferences were measured in triplicates to the nearest 0.5 cm with a measurement tape; for waist at the midpoint between the lower margin of the lowest palpable rib and the top of the iliac crest, and for hip at the widest portion of the buttocks^37^. Both measurements were conducted during end of expiration with the subject standing with feet close together and arms at the sides. Means from triplicate measurements of waist and hip circumference were used for calculations. Blood pressure was measured with a sphygmomanometer for manual readings with a cuff size of 12 × 35 cm (Welch Allyn, Skaneateles Falls, New York, U.S.) on the owners’ right arm after 5 min of resting in sitting position. Values < 130/85 mm Hg were considered normal^38,39^.

#### Dogs

Body condition score (BCS) of dogs was assessed by a veterinarian with specific expertise. Assessments were performed by observation and palpation of areas over the ribs, waist and abdominal line, according to guidelines from the World Small Animal Veterinary Association (WSAVA) based on the 9-point BCS scale validated by Laflamme^40^. According to this scale, a BCS of 1–3 represents underweight, 4–5 ideal weight, 6 slight overweight, 7 overweight and 8–9 represent obesity. Further, a clinical examination with specific focus on orthopedic status was performed by a veterinarian.

### Data processing and statistical analyses

**Figure 2:**
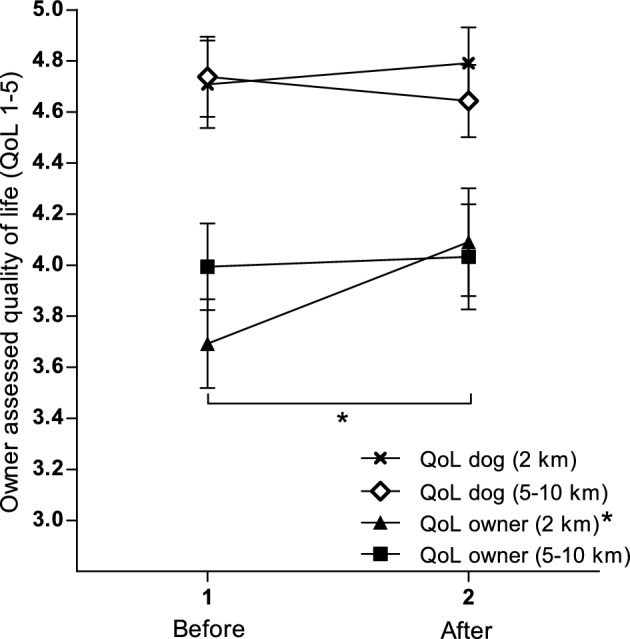
Line graph showing owner-assessed QoL in dog owners and dogs before and after the exercise intervention. A mixed model repeated measures analysis was used based on the two target distance groups; 2 km (n = 8) and 5–10 km (n = 14). Filled symbols represent mean ± SEM for owners for the two target distance groups and unfilled symbols represent the corresponding for dogs. The star (*) represents significant results for the whole cohort of owners and within group, respectively (*P* < 0.05). *QoL* quality of life.

**Figure 3:**
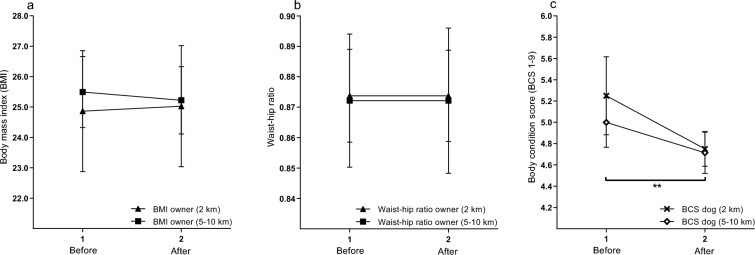
Line graphs showing (**a**) BMI in owners, (**b**) waist-hip ratio in owners and (**c**) BCS in dogs, before and after the intervention. A mixed model repeated measures analysis was used based on the two target distance groups; 2 km (n = 8) and 5–10 km (n = 14). Filled symbols represent mean ± SEM for dog owners and unfilled symbols represent the corresponding for dogs. Stars (**) represent significant results for the whole cohort of dogs (*P* < 0.01).

In this study, quantitative data were generated from body measurements and questionnaires. Microsoft Excel, SAS (SAS 9.4 Institute Inc., Cary, NC), and GraphPad Prism (GraphPad Prism 5.0 San Diego, CA) were used for data processing, statistical analyses and creation of figures. Data from participants that cancelled or dropped out during intervention was excluded. D'Agostino-Pearson omnibus normality test was used for testing of normal distribution of data, except in the mixed model repeated measures analyses where visual assessment of residuals was used. All data were normally distributed except for data on exercise time before the intervention and everyday physical activity after the intervention. Results are presented as mean value ± standard deviation (SD) or median and range (min–max), except for visualization of data in Figs. 2 and 3 where standard error of mean (SEM) was used. Paired analyses were two-tailed. Threshold for statistical significance was set to *P* < 0.05 in all analyses.

#### Data processing of “everyday physical activity”, “exercise” and “sedentary behavior”

For calculation of time spent in everyday physical activity, exercise and sedentary behavior, the middle value in the time interval for each answer category was used. Hence, for everyday physical activity and exercise, the middle value for “ < 30 min” was set to 15 min, the middle value for “30–59 min” to 45 min, etc. “0 min” counted as 0 min, “ > 300 min” as 300 min and “ > 120 min” as 120 min. For sedentary time, “Virtually all day” counted as 15 h and “Never” as 0 h. For calculations of proportion of dog owners fulfilling global recommendations for physical activity, WHO recommendations for adults aged 18–64 were used; at least 150–300 min of moderate-intensity aerobic physical activity; or at least 75–150 min of vigorous-intensity aerobic physical activity; or an equivalent combination of moderate- and vigorous-intensity activity throughout the week^41^. Everyday physical activity was here equated with moderate-intensity aerobic physical activity as defined by WHO^41^, and exercise was equated with vigorous-intensity aerobic physical activity^41^. An “equivalent combination” of moderate- and vigorous-intensity activity was equated with total physical activity load (also called “activity minutes”) per week, as described by Olsson et al.^42^. For calculation of total physical activity load (“activity minutes”), time spent exercising was multiplied by two to account for the proposed higher intensity and summed with the time spent in everyday physical activity^31^.

#### Paired statistical analyses and linear regressions

Wilcoxon matched-pairs signed rank test was used for comparisons of total physical activity load, everyday physical activity, exercise and sedentary time for the whole cohort of dog owners before and after the joint exercise program as all measurements were not normally distributed. A paired t-test was used for pre–post comparisons of blood pressure, waist and hip measurements as well as waist-hip ratio for the whole cohort of dog owners. Feeding routines and feeding-related behavior for the whole cohort of dogs was also analysed with a paired t-test. Associations between BMI in dog owners and BCS in their dogs were investigated with linear regressions.

#### Mixed model repeated measures analyses

For mixed model repeated measures analyses, data from participants were divided into two groups based on selected target distance: 2 km and 5–10 km. Mixed model repeated measures analyses were used for comparisons of QoL in dog owners and dogs, BMI and BCS as well as behaviors related to QoL in dogs. These comparisons were made for the whole cohort of dog owners and dogs before and after the intervention, as well as between groups (2 km and 5–10 km) and pairwise comparisons both before and after the intervention and between groups (interactions). Tukey–Kramer adjustments were used to correct for multiple comparisons in the mixed model.

#### Descriptive statistics

Data from additional questions on overall perceived changes after the intervention in the follow-up questionnaires (Fig. 1) were analyzed by descriptive statistics for the whole cohort of dog owners and dogs respectively. A mean value greater than 3 on the five-point Likert scale was interpreted as a perception of overall change.

## Results

### Study population

A total of 35 pairs signed up for the study. Six pairs cancelled after completing the baseline questionnaires, and an additional seven pairs dropped out during the exercise intervention. The remaining 22 pairs completed the whole study. Ten participants had a temporary break at some point during the joint exercise program, of which seven participants stated that the break was due to lighter injury or illness in themselves or their dog. Eight dog owners selected the target jogging distance 2 km and 14 dog owners selected a distance of 5–10 km. In the latter group, nine participants selected the distance of 5 km, four selected 7.5 km and one selected 10 km.

#### Dog owners

The mean age of the participants was 45 ± 12 years. Before the intervention, BMI ranged from underweight to obesity (16.5–33.2) according to the categorization by WHO^36^. Distribution of dog owner BMI is shown in Table 1.

**Table 1:** Baseline characteristics of participating dog owners (n = 22) and dogs (n = 22).

Dog owner data	Total number (n)	Proportion (%)
Gender		
Females	19	86
Males	3	14
Other	0	0
Age		
18–30 years	4	18
31–50 years	8	36
> 50 years	10	45
BMI		
< 18.5	1	5
18.5 – < 25	11	50
≥ 25 – < 30	3	14
≥ 30	7	32

Dog data	Total number (n)	Proportion (%)
Sex		
Intact females	7	32
Intact males	7	32
Neutered females	4	18
Neutered males	4	18
Age		
1–3 years	8	36
4–7 years	9	41
> 8 years	5	23
BCS		
1–3	1	5
4–5	14	64
6	6	27
7	1	5
8–9	0	0

*BMI*, body mass index, *BCS* body condition score.

#### Dogs

Dogs of 17 different breeds participated in the study. The mean age of dogs was 5 ± 3 years. On the 9-point BCS scale, dogs ranged from 3–7 (median 5) before the joint exercise program, i.e. slightly underweight to overweight. Distribution of BSC in dogs is shown in Table 1. Three dogs were included despite having owner-reported health issues such as mild joint disease, well-controlled hypothyroidism or allergy, as they were assessed by the examining veterinarian to be in a condition allowing participation in the study. No remarks associated with the exercise program were registered after the intervention.

### Outcome measures

#### Dog owner-assessed physical activity and sedentary time

There was a significant increase of both total physical activity load (“activity minutes”) and time spent on exercise in dog owners after the intervention compared to before, and a significant decrease of sedentary time (Table 2). After the intervention, all dog owners fulfilled global recommendations of a total physical activity load of at least 150 min per week (Table 3).

**Table 2:** Dog owner-assessed physical activity and sedentary time before and after the exercise intervention for dog owners (n = 22).

Dog owner-assessed physical activity and sedentary time	Before Mean/Median (Range)	After Mean/Median (Range)	*P-*value
Total physical activity load/”activity minutes” (minutes per week)	357/368 (105–540)	423/443 (225–540)	0.008
Everyday physical activity (minutes per week)	238/300 (75–300)	252/300 (75–300)	0.555
Exercise (minutes per week)	59/60 (0–120)	86/105 (0–120)	0.001
Sedentary time (hours per day)	7.5/6.5 (2–14)	6.1/5 (2–11)	0.039

**Table 3:** Proportion of dog owners (n = 22) fulfilling global recommendations^41^ for total physical activity load/”activity minutes”, moderate-intensity aerobic physical activity and vigorous-intensity aerobic physical activity before and after the intervention.

Descriptive statistics of dog owner-fulfilment of global recommendations of physical activity	Before proportion (%)	After proportion (%)
150 min of total physical activity load/”activity minutes” per week	91	100
150 min of moderate-intensity aerobic physical activity per week	77	82
75 min of exercise vigorous-intensity aerobic physical activity per week	50	73

#### Quality of life in dog owners and dogs

Data for QoL of dog owners and dogs is presented in Fig. 2. A significantly increased (*P* = 0.02) QoL in all dog owners was registered after the intervention (4.1 ± 0.7) compared to before (3.9 ± 0.6), a change that was driven by the 2 km group (*P* = 0.04). This improvement was supported by the mean of the additional follow-up question on perceived overall changes after the intervention (3.4 ± 1.3). Owner-assessed QoL in dogs was constantly high and did not change after the intervention (4.7 ± 0.5 versus 4.7 ± 0.5, *P* = 0.95). Neither was there any pre–post changes (*P* ≥ 0.83) in QoL in dogs within the two target distance groups. However, the additional follow-up question on overall changes in QoL of dogs indicated a perceived partial improvement of QoL after the intervention (3.4 ± 1.3).

#### Behaviors related to quality of life in dogs

There was no significant difference in behaviors related to QoL in the whole cohort of dogs after the intervention compared to before (*P* ≥ 0.10) in any of the nine question blocks (Supplementary Material 3). Neither was there any pre–post difference within the two target distance groups in any in the blocks, respectively (*P* ≥ 0.27). For eight of the nine question blocks, no overall changes in behaviors were perceived according to the additional follow-up questions. However, for the follow-up question on cardio fitness in the block “Vitality, activity and movement” a mean of 3.4 ± 1.5 was registered, indicating that dog owners did experience a partial improvement of cardio fitness in their dogs.

#### Dog owner motivation for joint exercise with their dog and self-acceptance of bodily appearances

A majority of owners (86%, n = 19) stated that their motivation for joint exercise with their dog had increased after the intervention, according to the additional follow-up question on motivation (4.1 ± 1.2). Ten respondents registered the maximal score of 5 for this question (“I totally agree”), and nine registered the number 4 (“I agree partially”). In the questionnaire on dog owner QoL, the question on self-acceptance of bodily appearances stood out, showing a significant increase (*P* = 0.03) in all dog owners after the intervention (3.5 ± 1.4) compared to before (3.1 ± 1.2). Forty-one percent of dog owners (n = 9) stated that their self-acceptance of bodily appearances had increased by choosing a higher score after the intervention.

#### Feeding routines and feeding-related behavior in dogs

No considerable changes were registered regarding feeding frequency, administered amount of feed or owner-assessed fullness after feeding after the intervention in comparison to before (Supplementary Material 3). Neither was there a significant change (*P* = 0.54) in the question block for feeding-related behaviors (such as appetite, tendency to steal or beg for food) after the intervention (3.5 ± 0.6) compared to before (3.4 ± 0.7), and no overall change in appetite was perceived according to the mean (2.1 ± 1.5) of the additional follow-up question in this section.

#### Body measurements in dog owners and dogs

Body mass index and waist-hip ratio in dog owners are shown in Fig. 3a,b. There was no significant change in BMI in all dog owners after the intervention (25.1 ± 4.6) compared to before (25.3 ± 4.7, *P* = 0.56), nor within the two target distance groups after compared to before the intervention, respectively (*P* ≥ 0.10). Neither were there any significant changes in waist-hip ratio in all dog owners after the intervention (0.87 ± 0.1) compared to before (0.87 ± 0.1, *P* = 1), nor in waist or hip circumferences (Supplementary Material 3). There were no significant pre–post changes within the two target distance groups for waist/hip measurements or ratio (*P* ≥ 0.27).

Body condition score in dogs is shown in Fig. 3c. A significantly reduced BCS (*P* = 0.008) in all dogs was registered after the intervention (4.7 ± 0.6) compared to before (5.1 ± 0.9.) There were no significant pre–post differences in BCS within the two target distance groups (*P* ≥ 0.12). Post-intervention BCS scores ranged from 4–6 (median 5), implicating that no dogs were longer scored as either underweight (BCS ≤ 3) or overweight (BCS 7) after the exercise program. There was no association between BMI in dog owners and BCS in their dogs, neither before the intervention nor after (*P* ≥ 0.37).

#### Blood pressure in dog owners

Systolic and diastolic blood pressure data for dog owners is presented in Supplementary Material 3. Mean values were below the defined cut-off for normal values both before and after the intervention, with no significant pre–post changes. Three dog owners reported that they were under medical treatment for hypertonia.

## Discussion

This unique pilot study of a joint outdoor exercise program for dog owners and their dogs showed that QoL in dog owners significantly increased and that BCS of dogs significantly decreased after the intervention. No changes in body measurements or blood pressure were registered in dog owners, but owner motivation for exercising together with their dog, as well as their self-acceptance for bodily appearances increased after the intervention.

Significant increases in self-assessed weekly total physical activity load and vigorous-intensity aerobic physical activity were registered by dog owners after the intervention, resulting in a 100% fulfilment of WHO global recommendations of physical activity from 2020^41^. Moreover, daily sedentary time was significantly decreased. Taken together, this indicates that the intervention increased physical activity in dog owners, which was stated by the Swedish Working Dog Association as one of the aims of the joint outdoor exercise program^25^. It should be noted that 91% of the dog owners fulfilled the recommendations for total physical activity load even before the intervention, which is in line with previous studies showing an association between dog ownership and higher levels of physical activity^7–10^, as the corresponding proportion for the Swedish population in 2021 was 67%^43^. No assessments of physical activity time in dogs were made, but the decrease in BCS in combination with the owner-perceived improvement in cardio fitness in dogs indicate that the exercise program did result in a corresponding increase of physical activity in dogs. Whether the current exercise program may constitute a sustainable way to increase physical activity time in dogs should be explore further, since previous research have indicated that lack of exercise is an emerging problem for our companion dogs^5,26,44^ and as such, successful strategies to increase their physical activity are warranted.

A significantly increased QoL was registered in all dog owners after the intervention, which supports previous research on the effect of physical activity on QoL^17,18,45^. It is also in line with previous findings on the positive effects of outdoor physical activity on different aspects of mental wellbeing^19–21^. The improvement in QoL in the current study was driven by participants who selected the shortest target distance (2 km), indicating that jogging even short distances on a regular basis can be beneficial for self-perceived wellbeing and that a joint active lifestyle should be encouraged even for people unable to exercise at a higher level. No changes were registered in overall QoL in dogs, nor in canine behaviors related to QoL. However, according to the additional follow-up question on perceived changes after the intervention, a slight improvement in QoL in dogs was still experienced. A novel aspect of the present study was that it did not focus on QoL in primarily overweight or obese dogs, as opposed to previous research^22,46^. In fact, 14 out of the 22 participating dogs in the present study were assessed as ideal weight (BCS 4–5) at the onset of the intervention. Thus, our results do not exclude the possibility that even normal weight dogs may benefit both physically and mentally from physical exercise, but that the questionnaires could not detect any subtle changes. In addition, a high owner-assessed overall QoL was registered for the dog population before the exercise intervention, leaving little room for improvement.

There were no significant changes in dog owner BMI or waist-hip ratio after the exercise intervention, neither for the whole cohort of owners nor on group level (2 km versus 5–10 km). It is noteworthy that an increase in acceptance for bodily appearances still was registered by 44% of dog owners, despite absence of changes in objectively assessed body measurements. It is possible that an impedance scale could have identified changes in body composition that were not detected with tape measurements or BMI calculations. However, it has been suggested that pronounced effects on body composition are not to be expected from exercise interventions without caloric restrictions^47–49^. Even so, there are contrasting studies that do in fact show substantial reductions of BMI and other body measurements as a result of isolated exercise interventions^50–52^. However, these studies are generally based on obese populations and exercise interventions lasting 12–14 weeks. At the outset of the present study, participants ranged from underweight to obesity with a mean BMI of 25.3 ± 4.7, therefore making a significant reduction of BMI on cohort level less likely. Also, the intervention lasted only eight weeks, which may be too short a period for effects on BMI to be expected. Mean blood pressure in dog owners was below the defined cut-off for normal values, both before and after the intervention, and showed no significant pre–post changes. According to previous research, physical activity does not have pronounced effects on blood pressure in normotensive individuals^48,53^, and thus a significant reduction of blood pressure on cohort level should not be expected in the present study.

In dogs, a significantly decreased BCS was registered, despite no considerable reported changes in feeding frequency or administered amount of feed. Here, it should be noted that assessment of body composition, BMI in humans and BCS in dogs, differ widely; while BMI is calculated from length and body weight^36^, BCS assessment is exclusively based on palpatory and visual hallmarks associated to total body fat content^40^. After the intervention, a majority of dogs were assessed as normal weight (BCS 4–5). Only two dogs were still assessed as slightly overweight (BCS 6) and no dog was assessed as overweight (BCS 7). In a previous three months’ intervention where overweight dogs were prescribed at least 30 min of daily exercise together with their owner, BCS decreased significantly^14^. However, other studies have concluded that physical activity as a tool for weight reduction in dogs has a non-significant effect on BCS compared to the effect of caloric restriction^16,54^. The positive result in the current study of a decreased BCS as an outcome of exercise without caloric restriction in slightly overweight dogs should be investigated further. Research including merely slightly overweight dogs currently is scarce, but studies by Lindåse et al. and Gille et al.^11,13^ indicate a high prevalence of slightly overweight individuals in the Swedish dog population, highlighting the need to include dogs from different weight categories in future studies.

There is another aspect to consider in the broader perspective. In general, standardized exercise programs used in research are designed to study the effects of physical activity on specific anthropometric and physiological variables^47–50^. In contrast, the joint exercise program from the Swedish Working Dog Association was originally designed to increase outdoor physical activity in dog owners and dogs, thereby improving health and wellbeing and promoting sustainable lifestyle changes in both parties^25^. In the current study, a vast majority (86%) of the dog owners stated that their motivation for joint exercise with their dog had increased after the intervention, suggesting a possible prospect of such lifestyle changes. As an active lifestyle has been shown to be positively associated with healthier dietary habits^55–58^, continued joint exercise might result in synergistic positive effects on overall health in a longer perspective. The specific reasons behind the increase in dog owner motivation for joint exercise are unknown, but might be due to the sense of companionship, support and security that the dog provided during the exercise sessions. It has previously been shown that the social support from a dog can aid in mutual weight loss programs for dog owners and dogs^12,27^. This points at the importance of considering the human-animal bond, a term defined as “the mutually beneficial and dynamic relationship between people and other animals that is influenced by behaviors that are essential to the health and wellbeing of both”^59^, in health research in humans and companion dogs. In addition, dog owners might have experienced exercise-induced health benefits in their dogs, i.e. ideal BCS, which they wish to maintain by continued joint physical activity. The increased QoL and self-acceptance for bodily appearances in owners may also be a ground for the strong motivation to continue to exercise with the dog^60^, and is in line with previous studies noticing a positive assessment of oneself as an effect of physical exercise^61–63^. Moreover, the fact that the joint exercise program was undertaken outdoors may be of relevance as Thompson Coon et al.^21^ has reported that motivation for repeated exercise is greater for outdoor physical activity compared to indoor activity.

## Study limitations

In the present pilot study, participating dog owners and dogs acted as their own controls, and the study was able to detect significant changes in QoL in owners and BCS in dogs comparing results from the eighth weeks’ intervention with base line. However, in future studies of joint dog owner-dog exercise, it is recommended to include both negative controls (dog owners and dogs) that do not take part in the exercise program and controls (humans) that take part in the exercise program but without dogs. Such set up could further deepen the findings and might also result in additional data on factors that may stimulate physical activity. All questions in the questionnaires were not validated, and a relatively low number of participants was included in this study. For future research, a larger number of participants is recommended, in order to detect changes that might not have been possible to identify in this study. Moreover, as dog owners voluntarily signed up for the joint exercise program, it cannot be excluded that the participants were more physically active and more motivated to exercise with their dog compared to the average dog owner even at the outset of the study. However, the high number of participants selecting the 2 km target distance might indicate that many in fact were not particularly physically active on a high level at the outset of the study. Moreover, there was no long-term follow up. This should be included in future studies, especially since the current study showed promising results regarding motivation for continued joint dog owner-dog exercise.

## Conclusions

This joint outdoor exercise program showed health benefits of a shared active lifestyle for both dog owners and dogs. The study indicates that an eight-week exercise intervention alone, with a target distance of at least 2 km twice a week, may be sufficient to significantly increase QoL and acceptance of bodily appearance in dog owners and change slightly overweight dogs into an ideal body condition. The increased motivation for continued joint exercise may suggest potential for long-term lifestyle changes. The importance of the human-animal bond as a success factor for increased mutual physical activity and health benefits in both dog owners and dogs is recommended to be studied in a more in-depth manner in future studies.

### Supplementary Information

Supplementary Information 1.

Supplementary Information 2.

Supplementary Information 3.

## Data Availability

The authors confirm that the data supporting the findings of this study is available within the article and its supplementary material, with the exception of dog owner raw data, since it contains information that may compromise the privacy of participating dog owners. This raw data is stored on local servers at the University of Agricultural Sciences, Uppsala, Sweden and is available in de-identified form from the corresponding author upon reasonable request.
