# Supplementary material for: A pilot study of a joint outdoor exercise program for dog owners and dogs
Source: Sci Rep. 2024 Jun 21;14:14321. doi: 10.1038/s41598-024-65033-0 (PMC11192721; doi:10.1038/s41598-024-65033-0)
Supplement: Supplementary file 3 — Supplementary Information 3. [file 41598_2024_65033_MOESM3_ESM.pdf]

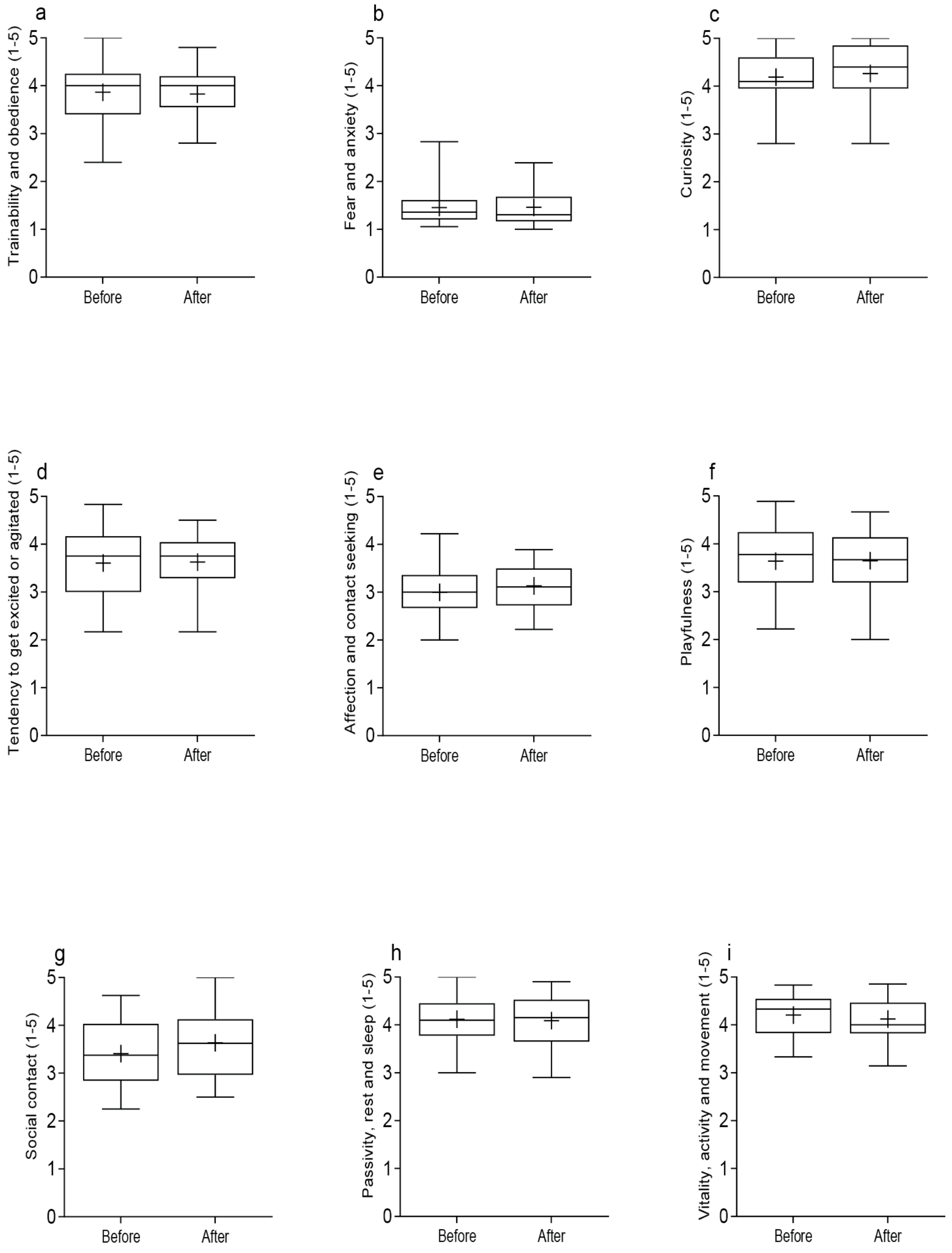

Supplementary Figure 1. Box plots of behaviors related to QoL in dogs. Boxes represent a) trainability and obedience, b) fear and anxiety, c) curiosity, d) tendency to get excited and agitated, e) affection and contact seeking, f) playfulness, g) social contact, h) passivity, rest and sleep, and i) vitality, activity and movement before and after the intervention for the whole cohort of dogs (n = 22). The boxes show the 25th–75th percentile, the line within the boxes shows the median, the plus sign shows the mean value and

the whiskers show maximum and minimum values. There were no significant pre–post changes in any of the question blocks.

**Feeding frequency in dogs**	**Before**<br>**Total number (n)**	**After**<br>**Total number (n)**
Fixed feeding hours	20	20
Sporadically	1	1
Free access	1	1
**Amount of feed according to feed package instructions (quantity/kg bodyweight)**	**Before**<br>**Total number (n)**	**After**<br>**Total number (n)**
Underfed	3	5
Normally fed	10	10
Overfed	2	2
Other	7	5
**Owner-assessed fullness after feeding**	**Before**<br>**Total number (n)**	**After**<br>**Total number (n)**
Full	17	16
Not full	3	5
Do not know	2	1

Supplementary Table S1. Feeding frequency, amount of feed and owner-assessed fullness after feeding in all participating dogs (n = 22) before and after the intervention.

**Dog owner body measurements**	**Before**<br>**Mean ± SD**	**After**<br>**Mean ± SD**	***P*-value**
**Waist circumference (cm)**	90.3 ± 12.0	89.8 ± 12.7	0.43
**Hip circumference (cm)**	103.4 ± 10.4	102.8 ± 10.0	0.12

Supplementary Table S2. Body measurements before and after the exercise intervention for the whole cohort of dog owners (n = 22)

**Dog owner blood pressure (Mm Hg)**	**Before**<br>**Mean ± SD**	**After**<br>**Mean ± SD**	***P*-value**
**Systolic value**	123.4 ± 13.2	123.7 ± 9.8	0.873
**Diastolic value**	73.1 ± 7.6	72.1 ± 7.5	0.480

Supplementary Table S3. Mean systolic and diastolic blood pressure measurements before and after the exercise intervention for the whole cohort of dog owners (n = 22)
